# GATE Validation of Standard Dual Energy Corrections in Small Animal SPECT-CT

**DOI:** 10.1371/journal.pone.0122780

**Published:** 2015-04-07

**Authors:** Sanghyeb Lee, Jens Gregor, Stephen J. Kennel, Dustin R. Osborne, Jonathan Wall

**Affiliations:** 1 Department of Electrical Engineering and Computer Science, University of Tennessee, Knoxville, TN 37996 USA; 2 Department of Radiology, Graduate School of Medicine, University of Tennessee, Knoxville, TN 37920 USA; 3 Department of Medicine, Graduate School of Medicine, University of Tennessee, Knoxville, TN 37920 USA; Institute of Automation, Chinese Academy of Sciences, CHINA

## Abstract

This paper addresses ^123^I and ^125^I dual isotope SPECT imaging, which can be challenging because of spectrum overlap in the low energy spectrums of these isotopes. We first quantify the contribution of low-energy photons from each isotope using GATE-based Monte Carlo simulations for the MOBY mouse phantom. We then describe and analyze a simple, but effective method that uses the ratio of detected low and high energy ^123^I activity to separate the mixed low energy ^123^I and ^125^I activities. Performance is compared with correction methods used in conventional tissue biodistribution techniques. The results indicate that the spectrum overlap effects can be significantly reduced, if not entirely eliminated, when attenuation and scatter is either absent or corrected for using standard methods. In particular, we show that relative activity levels of the two isotopes can be accurately estimated for a wide range of organs and provide quantitative validation that standard methods for spectrum overlap correction provide reasonable estimates for reasonable corrections in small-animal SPECT/CT imaging.

## Introduction

Dual isotope SPECT imaging using radioiodide is a useful technique for performing preclinical comparative effectiveness studies of biological agents, such as antibodies and peptides in individual animals. This technique significantly enhances comparison of reagents in vivo without interference from biological variability in the animal model. We have previously used dual-energy SPECT imaging of amyloid-reactive biological agents, ^125^I-labeled serum amyloid P component (SAP) and ^99*m*^Tc-labeled peptide, in a murine model of systemic visceral amyloidosis [1]. This is a very powerful technique for quantitatively comparing two biological radiotracers in an individual animal; however, to date we have used ^99*m*^Tc and radioiodide for this purpose, which involves two different methods of radiolabeling. Ideally, in comparative effectiveness studies both biological radiotracers should be labeled using the same technique, i.e., oxidative radioiodination of tyrosine side chain moieties [2]. This capability would allow quantitative comparison of novel amyloid-reactive peptides and the development of next generation reagents with improved binding properties [3, 4].

Dual energy imaging techniques are well established, e.g., ^99*m*^Tc-labeled perfusion agent and ^123^I-labeled neurotransmitter agents have been used in simultaneous acquisition for SPECT brain imaging [5]. ^99*m*^Tc and ^201^Tl are likewise used in rest and stress myocardial perfusion SPECT imaging [6]. The method for correcting spectrum overlap effects between these radionuclide pairs is relatively straightforward due to the energy gap in the emitted γ-photons. In contrast, the separation of radioiodine isotopes is more complicated because, although the primary γ-ray spectra of ^123^I and ^125^I are distinct and well-separated, the low energy spectra are identical with attenuation and scatter effects exacerbating the problem further. We show, and quantitatively validate with Monte Carlo techniques, that spectrum stripping methods used in gamma counters for separating the contributions from each isotope provides a sufficient estimation of the degree of spectrum overlap and can confidently be applied accurately to SPECT/CT imaging [7]. We further show that this simple method is quite robust with little deviation in the correction regardless of object size or relative activity concentrations with regard to typical mouse sizes and shapes.

Our work is centered around GATE-based Monte Carlo simulations for the MOBY mouse phantom [8, 9]. We use statistical system modeling to quantify the amount of spectrum overlap between ^123^I and ^125^I in the presence and absence of scatter and attenuation for different organs as well as different mouse sizes. Performance of the separation method is compared with conventional gamma counter tissue biodistribution measurements of two amyloid-reactive biological radiotracers in a mouse model of systemic, reactive amyloidosis [10].

## Results and Discussion

### Conventional gamma counter methods

Determination of the relative amounts of mixed isotopes in a sample was achieved using a gamma counter by first measuring the contribution of each radionuclide separately in the energy windows to be measured and applying spectrum stripping methods [7]. This method is routinely employed and has been used to determine the spectrum overlap from histological data [2, 5]. This technique provided a single correction factor that is typically used for the entire histology by subtraction of a percentage of counts from the low energy ^125^I window that are derived from ^123^I. For this study, the percentage determined from counting individual source standards in the gamma counter was 43%. The ^125^I data from gamma counting of samples of liver, spleen, kidneys, and heart were corrected for spectral overlap (spillover) by subtracting 43% of the counts measured in the ^123^I (high energy) window.

### Quantitative Analysis of spectrum overlap

**Fig 1:**
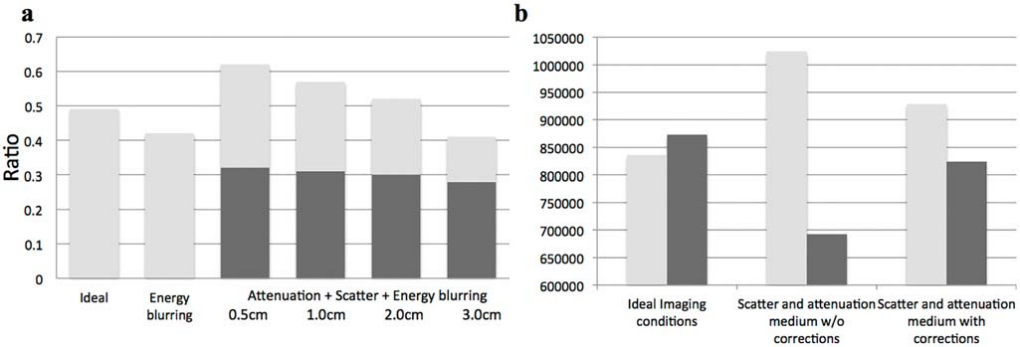
Stacked bar plots of energy ratios and relative activity measurements. a). Low-to-high energy ratio e123 under different imaging conditions. b). Total ^123^I activity in both high and low-energy windows.

**Fig 2:**
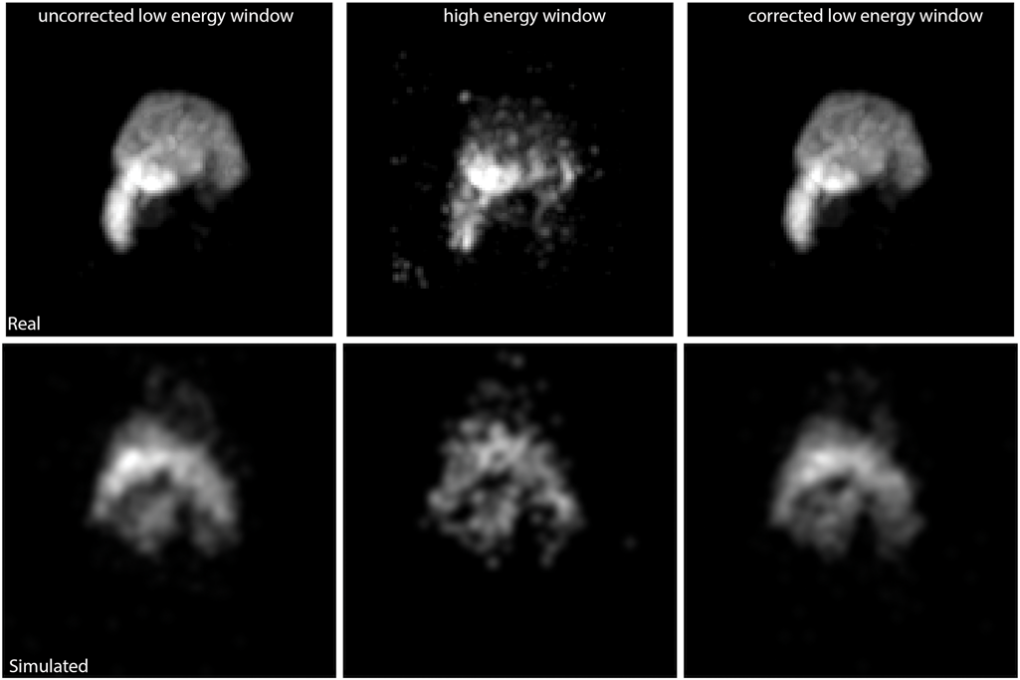
Comparison of real and simulated data. The top row shows real data for the uncorrected low energy window, high energy window, and the corrected low energy window. The bottom row shows the same information but for the simulated MOBY phantom data.

Under ideal imaging conditions with no scatter, attenuation, or energy blurring effects, the simulated ratio e123 was 0.49. When energy blurring was simulated, the ratio e123 decreased to 0.42. This expected result occurred because the energy blurring effect is stronger in the low energy window than in the high-energy window leading to truncation loss. When attenuation and scatter effects were included in the simulation, we observed that ratio e123 increased (Fig 1a). This occurs as a result of high-energy photons scattering into the low energy window. Interestingly, most of the scattering events were found to have taken place in the detector housing. In other words, the water sphere phantoms gave rise to a relatively small amount of scatter. Using the MOBY phantom under ideal imaging circumstances, the e123 ratio was found to be 0.49. However, when attenuation and scattering was introduced, ratio e123 dropped to 0.40. The total count in the high-energy window decreased by 20% because many of the high-energy photons were scattered (Fig 1b). In contrast, the total count in the low-energy window increased 22% as a result of the scattered high-energy photons being detected in the low-energy window. When scatter and attenuation corrections were both applied, an e123 ratio of 0.53 is obtained (Fig 1b). Visual comparison of simulated versus real data (Fig 2) show similar trends with each indicating less counts in the ^123^I high energy window and slight reduction in counts when the spectrum stripping correction methods were applied to the low energy window.

### Spectrum overlap estimation and correction

Amyloidosis in the AA mouse is most prevalent in the spleen, kidneys, liver, and heart; therefore we focused our analyses on these organs. Ratios of e123 were calculated for each of these organs in different sized MOBY phantoms (Table 1). Notably, the low-to-high energy ratio increased slightly (5–10%) as the size of the mouse phantom was increased. The activity detected in the high energy window was associated solely with ^123^I γ-photons, whereas the activity detected in the low energy window was a mix of ^123^I and ^125^I both γ-photons and x-ray photons. High and low energy ^123^I events are were statistically correlated, thus the low energy ^123^I component could be accurately estimated from the high energy counts. Separation of the mixed ^123^I and ^125^I activity signals detected in the low energy window could then be accurately achieved.

**Table 1:** Estimated low-to-high energy (e123) ratios for selected organs in different sized mice.

**Ratio Estimates for Mouse Organs**					
Organ	MOBY phantom size: radius (cm) /length (cm)				
	1.5/4	2/5	2/6	2.5/7	3/8
Spleen	0.54±0.005	0.54±0.008	0.54±0.004	0.56±0.003	0.57±0.008
Liver	0.57±0.004	0.58±0.006	0.58±0.005	0.6±0.005	0.62±0.005
Heart	0.53±0.004	0.54±0.004	0.54±0.008	0.54±0.004	0.56±0.007
Kidney	0.54±0.002	0.55±0.004	0.55±0.002	0.57±0.004	0.59±0.006

Simulations were carried out for different scanner and radiotracer concentration configurations to verify that the spectrum overlap effects can be suppressed using the simple ratio based correction method. The MOBY phantom was 4 cm wide and 6 cm long. The kidney was used as the source of emissions. For both the MGP and MWB collimators, the relative estimate error increased dramatically when scatter and attenuation medium were included in the simulation and predictably, decreased when appropriate corrections were applied (Table 2). When equal quantities (μCi) of ^123^I and ^125^I were simulated, the relative estimation error was 10% under ideal imaging conditions. When the amount of ^125^I was two-fold greater than ^123^I, the relative estimation error decreased because the spectrum overlap was reduced. Conversely, when the amount of ^123^I was present at two-fold the amount of ^125^I, overlap played a more dominant role due to the relative increase in ^123^I-derived x-ray photons and the scattered ^123^I high-energy γ-photons being detected in the low-energy window (Table 2).

**Table 2:** Relative estimation error for three different imaging conditions: Ideal (no scatter medium), scatter and attenuation medium without corrections, and scatter and attenuation medium with corrections.

Collimator	MGP			MWB		
Iodine Ratio	1:1	1:2	2:1	1:1	1:2	2:1
Ideal	6.4%±0.3%	3.8%±0.3%	11.3%±0.6%	6.8%±0.3%	4.6%±0.6%	14.5%±0.5%
w/o A+S	16.3%±0.6%	8.4%±0.4%	21.1%±0.4%	18.6%±0.7%	9.2%±0.5%	32.3%±0.6%
w/ A+S	7.1%±0.4%	3.6%±0.4%	11.3%±0.7%	11.2%±0.5%	5.9%±0.4%	19.2%±0.2%

## Materials and Methods

### GATE Model and Data Processing

**Fig 3:**
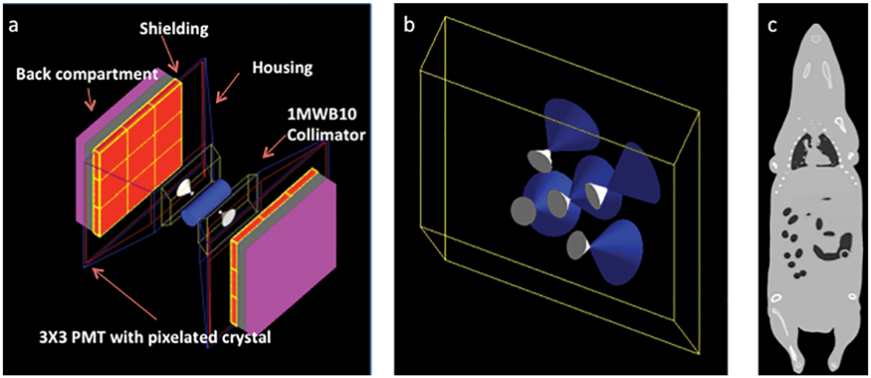
Illustrations of model components. a). GATE model of Siemens Inveon SPECT system configured with two MGP collimators. b). MWB collimator. c). MOBY attenuation phantom.

Spectrum overlap between ^123^I and ^125^I was quantitatively assessed by running GATE simulations for a selected set of imaging conditions. We used a validated GATE model of the Siemens Inveon multi-modality imaging platform (Fig 3a), which features a dual-head SPECT system with interchangeable collimators [11]. This model is available for download at the following hyperlink: GATE Model Inveon Download

Two tungsten collimators were used in the simulations. Collimator shape and material characteristics were modeled according to the manufacturers specifications in order to accurately account for resolution and other relevant data acquisition effects. The mouse whole-body collimator (MWB) has a five-pinhole configuration with 1.0 mm pinholes that supports whole body imaging of mice (Fig 3b). The mouse general purpose (MGP) collimator is a single pinhole, high-resolution imaging collimator with a 1.0 mm pinhole.

Simulated mouse data were generated using the MOBY phantom software (Duke University, Durham, NC) which features realistic organ shapes while maintaining the flexibility to model anatomical variations (Fig 3c) [9]. Liver, spleen, kidneys, and lung organs were simulated using the software and the resulting map of attenuation coefficients were converted to voxelized phantoms for use as the material in the realistic GATE model. ^125^I and ^123^I concentrations for each of the simulated organs were determined from real mouse data and the appropriate concentration distributed uniformly throughout each of the simulated organs for use as the source distribution input for the GATE model. Parameters used for the MOBY phantom are included as supporting information, S1 Data.

A 45 minute SPECT acquisition was simulated with the two detectors located respectively at 90 and 270 degrees at the start of simulation. Sixty projections were acquired with projection data acquired every 6 degrees over a 360-degree gantry rotation. For the MGP collimator, a 25 mm radius of rotation was used. For the MWB collimator, a 30 mm radius of rotation was used.

The Inveon Acquisition Workplace (IAW) software version 1.5 (Siemens Medical Solutions USA, Inc., Knoxville, TN) was used to acquire and reconstruct tomographic images from the simulated phantom projection data and real animal images. Using this software, CT-based attenuation correction and dual-energy window scatter correction [12] was applied to data from both the high and the low energy windows. Data were also reconstructed without attenuation and scatter correction to determine its impact on calculated ratios.

**Fig 4:**
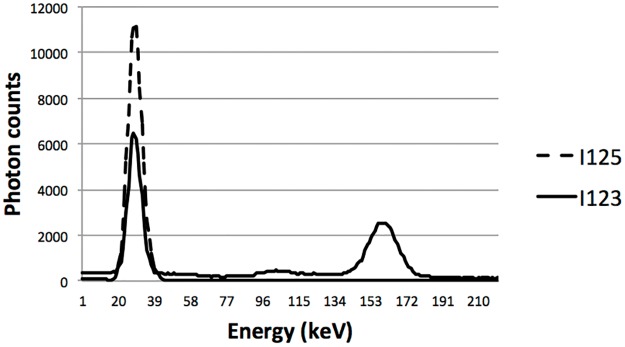
Energy spectra of ^123^I and ^125^I for the MGP collimator.

Each iodine isotope was modeled using their three primary energy peaks. All other energy peaks were omitted. For ^123^I, the energy peaks used were 528 keV (1.1%), 159 keV (48.4%), 31 keV (9.3%), and 27.5 keV (41.1%). For ^125^I, the energy peaks used were 35.5 keV (4.5%), 31 keV (17.6%), and 27.5 keV (77.9%). One detector head was configured to have an energy window of 148170 keV with an energy blurring of 11% while the other detector head was set to have an energy window of 2545 keV with an energy blurring of 28%. Fig 4 shows the two spectra as detected with the MGP collimator.

### Spectrum Overlap Estimation and Correction

Data were separated into high and low energy windows represented as HI and LO, respectively. Each window contains all the counts acquired within that window for a given isotope where we use the terminology HI123 and LO123 referring the spectrum based ^123^I activities, and LO125 for the low energy ^125^I activity. Under ideal circumstances, where attenuation, scatter, and effects such as detector induced energy blurring can be ignored, it is well-known that the low-to-high energy ^123^I ratio given by:

$$\begin{array}{c} e_{123}=LO_{123}/HI_{123} \end{array}$$ (1)

can be used to estimate the ^125^I activity in the low energy window using the formula:

$$\begin{array}{c} \left(LO_{125}=LO-e_{123}HI\right) \end{array}$$ (2)

To investigate how each physical and detector interaction influences the low energy photon spectrum overlap, GATE simulations were carried out for the following scenarios: (1) ideal imaging situation (assumption of no attenuation or scatter), (2) energy-blurring was considered, and (3) energy-blurring, attenuation, and scatter effects were all taken into account and the source was located inside different sized water spheres with radius varying from 0.5 cm to 3.0 cm. Each simulation was designed to represent 200 μCi ^123^I in a uniform sphere (radius = 3.91 mm) with the activity distributed over a 250 mL volume. The effect of different activity concentration ratios of ^123^I to ^125^I were also examined in our simulations with this uniform spherical phantom. The ratios simulated were 1:1, 1:2, and 2:1 for ideal conditions (no attenuation or scatter), attenuation and scatter simulated but not corrected, and attenuation and scatter simulated but corrected for in reconstruction.

GATE simulations were ran to estimate generic organ based e123 ratios for different sized mice with length varying from 4cm to 8cm. The e123 ratios were pre-computed for different organs under various circumstances using the MOBY phantom output in the GATE simulation. These pre-computed e123 ratios were then used to estimate the amount of spectrum overlap effects in the low energy window of real mouse data using Eq (2). The accuracy of the correction was captured by the difference between the estimated and the actual ^125^I activity measured relative to the latter. We refer to this as the relative estimation error given by:

$$\begin{array}{c} r_{125}=\left(Actual_{125}-Estimate_{125}\right)/Actual_{125} \end{array}$$ (3)

where Actual denotes the actual number of I photons detected and Estimate denotes the estimated number computed using Eq (2).

Each simulation was repeated five times to acquire a mean value for each result. Standard deviations were calculated for each series of simulations and compared. The coefficient of variation (COV) was calculated for series of simulations. COVs of 20% and 10% were deemed acceptable for individual and entire population averages, respectively.

### Animal Studies

This study was carried out in strict accordance with the recommendations in the Guide for the Care and Use of Laboratory Animals of the National Institutes of Health. The protocol was approved by the Institutional Animal Care and Use Committee (IACUC) of the University of Tennessee. Animals for this study are housed in our IACUC approved dedicated animal facility.

To compare our models with standard methods, we acquired SPECT and CT scans of three mice with systemic AA amyloidosis using an Inveon trimodal (PET/SPECT/CT) imaging system. Each mouse was injected with 145 μCi of ^125^I-protein 1 and 45 μCi of ^123^I-protein 2. After the appropriate uptake time (2 h after injection of the second protein), the mice were euthanized by isoflurane overdose and prepared for SPECT/CT imaging. Sixty projections were acquired at 6-degree intervals over 360-degrees. A full energy window from 0-300 keV was acquired and subsequently histogrammed into three energy windows for both detector heads. For detector 1, with voltage and gain set to acquire ^123^I emission data, we used 127–143keV, 143-175keV, and 175-191keV windows. For detector 2, with voltage and gain set to acquire ^125^I emission data, we used 15-25keV, 25-45keV and 45-55keV windows. SPECT and CT scans were used to estimate e123 ratios using Eq (1).

After image acquisition, the mice were sacrificed, necropsied, organs harvested with a small piece placed in a tared vial, and counted in a Wizard 3 1480 gamma counter (Perkin Elmer, Waltham, MA). The amount of ^123^I and ^125^I radioactivity in each sample was measured. Spectrum overlap corrections for this technique were applied by using pure samples of both isotope and calculating the low-energy component of the ^123^I. These data were also used to calculate the e123 ratios using these standard methods. Because of differences between output of the imaging system and gamma counter, data acquired from both systems were corrected to provide units of percent injected dose per gram (%ID/g) decay corrected to the animal injection time.

## Supporting Information

S1 Data Data parameters file for MOBY phantom generation. (ZIP)

S1 ARRIVE Checklist Completed ARRIVE checklist. (PDF)
